# “Living Inside the Walls”: Systematic Review and Qualitative Meta-Synthesis

**DOI:** 10.2196/78142

**Published:** 2026-04-17

**Authors:** Weitong Li, Ziqi Mei, WenJing Tu, Yulei Song, Yayi Zhao, Yamei Bai, Guihua Xu

**Affiliations:** 1 School of Nursing Nanjing University of Chinese Medicine Nanjing China; 2 Department of Psychology Academy of Advanced Interdisciplinary Studies Wuhan University Wuhan China

**Keywords:** social isolation, meta-synthesis, LTCF, long-term care facility, older people, qualitative research, systematic review

## Abstract

**Background:**

Social isolation is a prevalent issue among older people in long-term care facilities (LTCFs), with profound negative impacts on their quality of life and mental health. However, the authentic experiences and underlying mechanisms of social isolation among older people in LTCFs remain understudied. A nuanced understanding of these experiences is essential for designing targeted nursing interventions.

**Objective:**

This study aimed to systematically review and synthesize qualitative evidence to explore the experiences of social isolation among older adults in LTCFs and analyze them in terms of causes, mechanisms, outcomes, and strategies.

**Methods:**

A systematic review and qualitative meta-synthesis were conducted. We searched PubMed, Web of Science, Embase, PsycInfo, Cochrane Library, CINAHL, Scopus, Chinese Biomedical Literature Database, CNKI, Wanfang Data, and Weipu (VIP) with no restriction on the start year, up to February 2025. Two reviewers (WL and ZM) independently screened studies, extracted data using a Microsoft Excel spreadsheet, and assessed study quality with the Joanna Briggs Institute Critical Appraisal Checklist for Qualitative Research. Qualitative data synthesis was performed following Thomas and Harden’s thematic and content analysis method, including line-by-line coding of participant quotes, organizing codes into descriptive themes, and developing analytical themes.

**Results:**

A total of 4856 papers were retrieved through systematic searches, and 14 qualitative studies met the inclusion criteria. Four overarching analytical themes were identified: (1) root cause: person-environment mismatch (encompassing 4 interrelated dimensions: imbalance between declining individual capacity and insufficient environmental support, negative self-perception paired with inadequate positive environmental feedback, personal biases conflicting with homogeneous institutional social ecology, and disrupted social roles due to rigid institutional management); (2) overt behavioral patterns: proactive isolation, defensive isolation, and adaptive isolation; (3) inner emotional experiences: feelings of confinement, sorrow, and weariness; and (4) strategies for restoring person-environment match: active engagement in interaction, rational acceptance of coexistence, and passive avoidance and withdrawal. Notably, person-environment mismatch was confirmed as the core mechanism driving the onset and persistence of social isolation in this population.

**Conclusions:**

Person-environment mismatch is the fundamental driver of social isolation among older adults in LTCFs. Interventions should prioritize restoring person-environment alignment—including optimizing physical environments, reconstructing supportive social environments, and enhancing psychological empowerment. Future studies should focus on developing tools to quantify person-environment matching degrees in LTCFs and conduct longitudinal evaluations of targeted interventions to further reduce social isolation and promote active social participation among older people.

## Introduction

**Figure 1:**
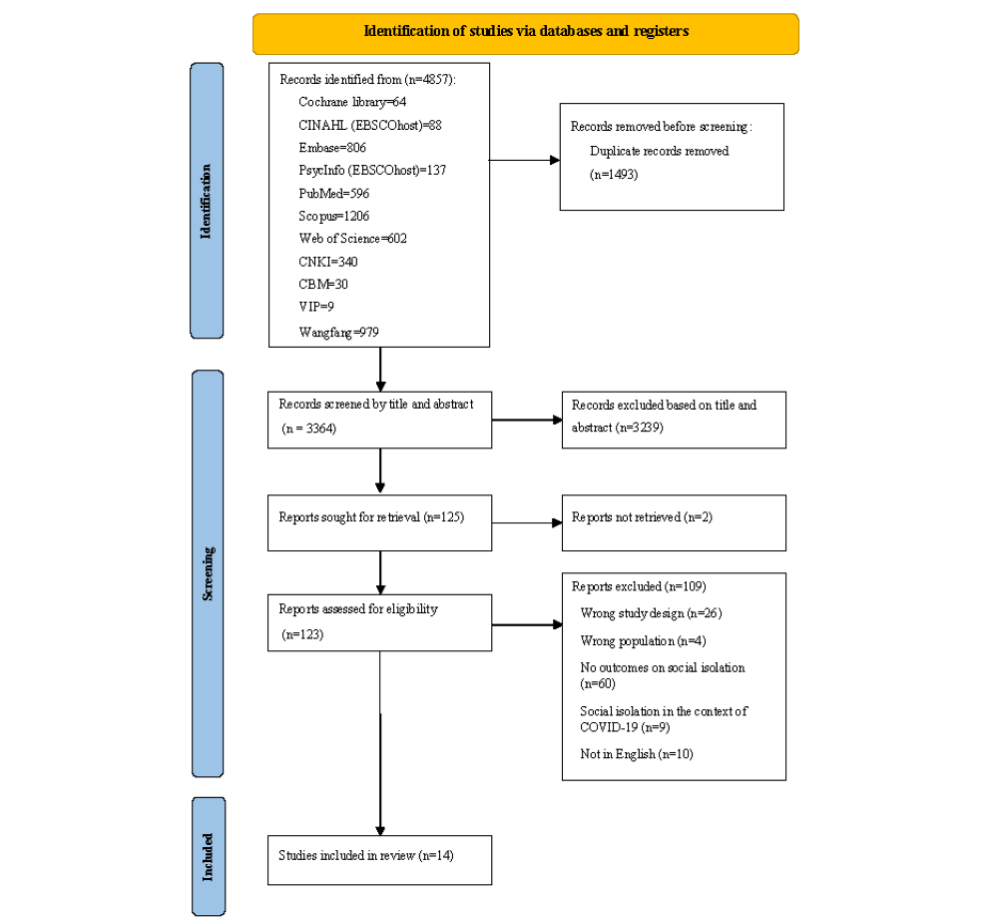
PRISMA (Preferred Reporting Items for Systematic Reviews and Meta-Analyses) flowchart.

Population aging is an increasingly prominent global issue, with projections indicating that the number of adults aged 65 years and older will exceed 1.6 billion by 2050—more than double the current figure [1]. Against the backdrop of increasingly declining birth rates and an aging population, faced with practical challenges including family downsizing, regional separation, and a scarcity of community care resources, long-term care (LTC) is in high demand among older people. Given these circumstances, more families are choosing to place older relatives in long-term care facilities (LTCFs). The core mission of LTCFs is to support older adults in maintaining maximum independence, rather than providing services that replace their existing or potential abilities, undermine functional capacity, and increase care dependency [2]. Nevertheless, despite providing essential safety guarantees and professional care services, the inherent institutional attributes of LTCFs—marked by standardized structured environments, rigid administrative regulations, and centralized care protocols—may unintentionally exacerbate the risk of social isolation among older people [3]. To contextualize the focus of this study, a clear distinction is drawn between social isolation and loneliness: social isolation denotes an objective lack of meaningful social contacts and high-quality interpersonal relationships, while loneliness refers to a subjective emotional response to unmet interpersonal needs [4-6].

Social isolation represents a significant potential risk faced by older adults in LTCFs of older people care [7]. Evidence suggests that LTCFs do not adequately provide comprehensive care services suited to the diverse requirements of older people [8]. In LTCFs, tight space-time management standards restrict the timing, place, and manner of older individuals’ daily activities. Due to understaffing and severe workloads, caregiving staff devotes the majority of their time and energy to satisfying older people’s fundamental physical requirements [9], such as aid with daily living and medical care. In such settings, compared to those living in the community and at home, older people in LTCFs often lack meaningful social engagement, adequate sensory stimulation, and positive interpersonal relationships, and thus experience a far higher rate of social isolation [10,11]. A number of studies have shown that the social network of older people, as an important social resource, is closely related to their health and quality of life [12,13]. Older people in LTCFs who experience social isolation face an increased risk of mortality, higher morbidity, depression, and cognitive decline, and they are also more prone to physical pain and psychological distress [14]. Meanwhile, the deterioration in physical function makes older people in LTCFs more worried, worsens social isolation [15], and develops a vicious cycle, which may lead to a greater burden of institutional care and have negative societal impacts.

In actuality, for older people facing the risk of reduced social networks and increased social isolation, increasing social integration of older people is of vital importance, as it could alleviate the devastating sense of isolation and loneliness from LTC nursing and improve the quality of life of older people [16]. Despite decades of research on social isolation, most studies on social isolation of older people have focused primarily on community-dwelling older adults [17]. However, social isolation among older people in institutionalized care settings may differ significantly from that of older people living in communities in terms of mechanisms, manifestations, and effects [18]. Due to factors such as traditional cultural beliefs and weakened social functions of older people, with the aim of not burdening their children and reducing family burden, they may hide their emotional needs to some extent after entering in LTCFs, resulting in hidden and difficult to dynamically identify symptoms of social isolation. At the same time, due to management needs, LTCFs may encounter stricter institutional rules, limited environmental support, and reduced social network relationships [19]. Because of this group’s unique physical functions, cognitive status, and social roles, the complexity of personal and environmental circumstances increases their risk of social isolation, making it more difficult to overcome through regular social support networks. However, older people living in the community could usually keep certain social roles and engage in specific social activities to interact with the outside world through their existing relatives, colleagues, and neighbors [20]. As a result, moving the study focus to older people in LTCFs could improve their quality of life, lower medical expenditures, and lessen the burden of social care for older adults [21]. This not only broadens the situational relevance of social isolation theory but also provides a practical foundation for creating more tailored intervention tactics.

Given the institutional characteristics of LTCFs and the objective nature of residents’ social isolation, the person-environment fit (P-E fit) theory provides the core theoretical framework for explaining the underlying mechanisms of this phenomenon [22]. This theory focuses on the dynamic interaction between individuals and their environments, stressing that the alignment between personal needs or attributes and environmental resources or provisions determines adaptive outcomes [23]. Specifically, when older people’s core needs (autonomy, social engagement, and dignity) match LTCFs’ resources, norms, and support, they tend to maintain active social participation and well-being. Conversely, standardized care norms may suppress social initiative, and insufficient interaction opportunities fail to meet diverse needs. Such a mismatch may further induce psychological distress or physical health decline. From the P-E fit perspective, LTCF residents’ social isolation can thus be viewed as a negative outcome of the imbalance between older people’s personal resources and the environmental resources provided by the institution. However, this research offers a limited understanding of how older adults in LTCFs experience social isolation while neglecting older adults’ subjective experiences of this mismatch. Key questions remain: How do LTCF environments reshape residents’ social experiences? How do they make sense of, interpret, and respond to this sense of “isolation”? Answering these requires targeted qualitative exploration—laying the foundation for this study.

Based on the results of systematic sorting and comparison, this study opted to use qualitative meta-synthesis as the research approach [24]: (1) social isolation among LTCF residents differs in its nature and manifestations from that among community-dwelling older people; it differs significantly in formation mechanisms, manifestations, and influencing variables. Through rigorous literature screening and quality assessment, qualitative meta-synthesis can not only systematically integrate scattered findings from existing qualitative research and connect fragmented evidence (environmental constraints, personal perceptions, and interaction patterns) but also reveal the unique formation mechanism of social isolation among LTCF-dwelling older adults via “reinterpretation” and “theoretical synthesis,” thereby better addressing the uniqueness and innovation of this study [25]. (2) This study uses the P-E fit hypothesis to explain how environmental-person mismatch in LTCFs causes social isolation among older adults. Most current qualitative research focuses on 1-sided influencing factors, while qualitative meta-synthesis can identify qualitative evidence of “individual—environment—interaction” in existing studies, revise and refine the P-E fit theory framework for closed settings, and clarify the key mechanisms of social isolation in LTCFs and the specific forms of “mismatch” [26]. The need to deepen the P-E fit theory arises from the fundamental differences between LTCF residents and community-dwelling older adults [27]. Qualitative meta-synthesis could strengthen this theory to explain the formation mechanism of social isolation in LTCFs and potential solutions, thus promoting a systematic understanding of the phenomenon.

This review aimed to identify the experiences of social isolation among older people in LTCFs and analyze them in terms of causes, mechanisms, emotional experiences, and coping strategies. The findings of this study will inform the design and implementation of targeted interventions to alleviate social isolation and improve the well-being of this population.

## Methods

### Overview

We conducted a systematic review and qualitative meta-synthesis (PROSPERO CRD42024591240) and report the findings in accordance with the PRISMA (Preferred Reporting Items for Systematic Reviews and Meta-Analyses) (Multimedia Appendix 1) [28] and the ENTREQ (Enhancing Transparency in Reporting the Synthesis of Qualitative Research) statement [29] (Multimedia Appendix 2). We chose this study approach because qualitative meta-synthesis is useful for generating theoretical and conceptual knowledge, informing therapeutic interventions and research programs, and improving the clarity, comprehensiveness, and transparency of qualitative systematic reviews [30].

### Search Strategy

We conducted a comprehensive electronic search of English and Chinese databases, including PubMed, Web of Science, Embase, PsycInfo, the Cochrane Library, CINAHL, Scopus, the Chinese Biomedical Literature Database, CNKI, Wanfang Data, and Weipu (VIP). The search included qualitative studies exploring social isolation experiences among older adults receiving LTC, with no start date restriction and a cutoff date of February 2025. According to the PICOS (population, intervention, context, outcome, study design) framework recommended by the Joanna Briggs Institute (JBI) Centre for Evidence-Based Healthcare in Australia, 4 key concepts were used: older adults, social isolation, LTC, and qualitative research. A comprehensive search was conducted for each concept using a combination of MeSH terms and free-text keywords. A backward citation search was also performed for all identified studies. For detailed examples of our search strategy, see Multimedia Appendix 3.

### Inclusion and Exclusion Criteria

The PICOS model recommended by the JBI Evidence-Based Healthcare Center in Australia was used to determine the inclusion and exclusion criteria for the study. The inclusion criteria were as follows: (1) population (P): older people aged 60 years and older who are receiving LTC; (2) intervention (I): the causes and lived experiences related to social isolation among older adults receiving LTC; (3) context (C): residential facilities providing LTC for individuals who require assistance with daily living activities and have specific health and care needs, such as nursing homes or integrated medical and nursing care facilities; (4) outcome (O): the manifestations, impacts, and coping strategies of social isolation; and (5) study design (S): qualitative research or mixed method studies (only the qualitative component was included), using methodologies such as phenomenology, grounded theory, or ethnography.

The exclusion criteria were as follows: (1) studies where social isolation was not the primary research phenomenon but only a subtheme; (2) social isolation caused by lockdowns or isolation measures during the COVID-19 pandemic; (3) literature not available in English or Chinese; (4) full texts not accessible; (5) duplicate publications or studies with incomplete data; and (6) conference abstracts, commentaries, or reviews.

### Study Selection and Data Extraction

Search results were entered into EndNote (version 8; Clarivate Analytics), then exported to Covidence after duplicates were removed. Five reviewers (WL, ZM, WT, YS, and YZ) independently screened titles and abstracts, with full-text reviews conducted in duplicate (2 reviewers [WL and ZM] per paper) to identify eligible studies. Eligible data were retrieved and entered into a Microsoft Excel spreadsheet, which included (1) general study and sample characteristics, (2) methodology, (3) participant quotes, and (4) themes and findings. Throughout, conflicts were settled through discussion and judgment by a third reviewer (YZ).

### Appraisal of Methodological Quality

The JBI Critical Appraisal Checklist for Qualitative Research, with 10 items, guided the evaluation of the quality of the included publications [31]. Each publication was examined by 2 researchers (WT and YS) who worked separately. Disagreements were also resolved through discussion and further evaluation. Inclusion needed a specified minimum of “yes” in 6 domains. All included studies clearly outlined their objectives and provided a thorough explanation of how the research was conducted in alignment with these objectives. Two studies reported all items recommended by the JBI-Qualitative Assessment and Review Instrument tool.

### Data Synthesis

This study adopts a systematic review and thematic synthesis approach focusing on qualitative research. In the case of mixed method studies, only the qualitative data were included for analysis and integration to maintain methodological coherence. This study followed the three-stage thematic synthesis process proposed by Thomas and Harden [25]: (1) line-by-line coding of relevant texts: all descriptions related to the social isolation phenomenon among older people in LTCFs were compiled and coded; (2) organization of codes into descriptive themes: new descriptive themes were created by comparing the data from the first phase (eg, boredom and rational acceptance of coexistence), with connections clarified, similar themes merged, and grouped coding applied; and (3) development of analytical themes: the descriptive themes from the previous stage were re-examined, and similar descriptive themes were classified into a comprehensive outcome (the analytical themes included causes, manifestations, feelings, and coping strategies of social isolation). The synthesis analysis was conducted by 2 researchers (YS and YB), with a third experienced independent reviewer (GX) coordinating the thematic analysis. The entire research team carefully reviewed the data analysis process to ensure consistency in the interpretation of results and adequacy of the analytical themes.

### Rigor, Trustworthiness, and Reflexivity

#### Rigor

This study used a comprehensive search strategy across 11 databases, rigorous quality appraisal using JBI criteria, and consistent data analysis following Thomas and Harden’s [25] 3-stage thematic synthesis. Two researchers (WL and ZM) independently conducted screening, data extraction, and coding, with disagreements resolved through discussion and third-reviewer (YZ) adjudication, and the entire research team reviewed the coding and theme development process to ensure consistency and adequacy of the synthesized results.

#### Trustworthiness

Drawing on Lincoln and Guba’s [32,33] criteria for establishing trustworthiness in qualitative research, we implemented four strategies: (1) credibility: prioritizing participants’ direct quotes over authors’ interpretations to ensure that findings authentically represent older adults’ experiences. Member checking was indirectly achieved by verifying that the synthesized themes were consistent with the original findings of the included studies, with the research team discussing and confirming the alignment between coded data and thematic interpretations; (2) transferability: providing thick descriptions of study contexts (settings, participant characteristics, and care environments) to enable readers to assess applicability to other LTCF settings; (3) dependability: maintaining an audit trail documenting all analytical decisions, data extraction spreadsheets, coding frameworks, and theme development processes; this trail enables other researchers to replicate the study process and verify the consistency of the results; and (4) confirmability: 2 independent researchers (WL and WT) performed data extraction and coding, with a third reviewer (YS) resolving discrepancies to reduce bias. Thematic synthesis was guided by the P-E fit theory, and the research team repeatedly compared synthesized themes with original data to ensure that conclusions were data-driven rather than based on prior assumptions.

#### Reflexivity

The research team comprised nursing and psychology researchers with diverse backgrounds in geriatric care, institutional management, and qualitative methods. Acknowledging that team members’ clinical experiences and cultural contexts (Chinese health care settings) might introduce bias, we implemented three reflexive practices [34]: (1) maintaining a reflexive journal to document preconceptions and analytical decisions, with explicit documentation during thematic synthesis of instances where personal experiences or disciplinary backgrounds could influence interpretation; (2) conducting peer debriefing with external qualitative experts not involved in data analysis—an independent researcher specializing in social isolation reviewed the final thematic framework, providing feedback on potential biases to ensure that findings were objective and data-grounded; and (3) explicitly bracketing prior assumptions during coding via iterative comparison of codes against raw data. While 2 cofirst authors (WL and ZM) led data analysis, multidisciplinary perspectives from psychology and the older people care management team members counterbalanced individual interpretations.

## Results

### Overview

A total of 4856 studies were identified via systematic database and hand searches. Of these, 123 underwent full-text review, and 14 qualitative studies involving 210 older people were ultimately included in the meta-synthesis. The screening process is shown in Figure 1. The studies (2006-2023) were conducted in various regions, including China (n=7), the United Kingdom (n=3), Ireland (n=1), New Zealand (n=1), Australia (n=1), and Finland (n=1). The research settings included nursing homes, social welfare institutions, and hospitals. In studies that clearly report the sex of participants, 94 female of 148 (64%) participants were included, with a relatively lower proportion of male participants. However, the study analyses were based on the entire study population of older people. In total, 6 studies reported the duration of LTC, with a minimum duration of 10 weeks, while the remaining studies did not provide information on the length of care. A variety of qualitative methodologies were used, with mixed methods designs being the most common, with data collection methods primarily relying on semistructured interviews and participant observation. The main findings of this review are presented in Table 1.

**Table 1:** Characteristics of included studies.

Authors	Location	Aim	Participants	Age (years) of older adults	Settings	Duration	Research design	Data collection methods	Main results or themes
Bartlett [35]	United Kingdom	To explore how men with dementia experience, and deal with, nursing home life	1 older adult with Alzheimer disease	65-84	Nursing home	≥5 months	Case study	Semistructured interview	Experiences of social exclusion Aligning self with other men Aligning self with masculine behaviors
Buckley and McCarthy [36]	Ireland	To explore the lived experience of social connectedness as perceived by cognitively intact older adult residents	10 older people with scores greater than 6 on the Mini-Mental State Examination	71-99	Urban long-term care facility	≥1 year	Qualitative phenomenological design	Semistructured in-depth interviews	Superficial relationships Substitution Outside world connection Mental ability Attitudes or actions of caregivers Feelings of isolation
Cook et al [37]	United Kingdom	To understand the difficulties that residents with sensory impairments face when interacting with others	Study A: 8 older adults; study B: 18 older adults	Study A: 52-95; study B: 70-100	Nursing care home	Study A: 1.5-6 years; study B: not specified	Study A: hermeneutic inquiry; study B: constructivist study	Study A: interviews; study B: semistructured interviews, participant observation, and resident focus group interviews	Being a member of the resident community Getting to know other residents and supporting each other Maintaining interaction and developing relationships with fellow residents
Goll et al [38]	United Kingdom	To explore the relationship between social participation and social identity among lonely older adults living independently in London	15 older adults, all of whom reported some form of illness or disability	62-100	Urban voluntary sector organizations situated	Not specified	Thematic analysis	Semistructured interview	Overt barriers Responses to barriers Social fears Fear of losing preferred identities
Li and Bai [39]	China	To explore the current situation of social isolation among disabled older adults in older people care institutions, the underlying mechanisms, and its impact on their physical and mental health	14 older adults with mild to moderate disabilities	≥65	Nursing home	Not specified	Qualitative research	In-depth interview method	From spatial isolation to disconnection from external society From spatial isolation to disconnection from internal society From social disconnection to perceived isolation
Li [40]	China	To explore the current situation, challenges, and causes of social isolation among partially disabled older adults in institutional care	22 older adults with partial disabilities	≥65	Nursing home	≥6 months	Mixed methodological research	Semistructured in-depth interviews; participant observation method	Social isolation of semidisabled older people residents in X institution Problems caused by social isolation among semidisabled older people residents in X institution Causes of social isolation among semidisabled older people residents in X institution
Mu and Yin [41]	China	To understand the current situation and characteristics of the breakdown and reconstruction of social networks among older adults in nursing homes in southwestern Hubei	14 older people with clear thinking and articulate speech	65-87	Social welfare institute	≥6 months	Qualitative research	Semistructured in-depth interviews; participant observation method	Breakdown and reconstruction of social networks among older people residents in H social welfare institution Characteristics of social network breakdown and reconstruction among older people residents in H social welfare institution
Neves et al [42]	Australia	To better understand how frail older people experience social isolation and loneliness	22 residents experiencing (or at risk of) loneliness and/or social isolation, identified by staff	65-95	Care home	≥10 weeks	Qualitative research	Semistructured interviews; participant observation method	Meanings and contexts of loneliness and social isolation Loneliness as relational Loneliness as oldering Loneliness as a personal trouble Loneliness as a sickness Coping and protective strategies
Nielson et al [43]	New Zealand	To explore how residents of a commercially operated housing and care retirement complex experience inclusion and exclusion and their sense of community within a diverse environment of fit and frail residents	12 cognitively competent older adults	70-85	Commercially operated housing and care retirement complex	2.5-6 years	Ethnographic approach	Semiformal interviews; participant observation method; artifact method	Motivations and expectations Building social connections through participation Social group membership and belonging Health decline and social change Health decline and stigma
Pirhonen et al [44]	Finland	To shed light on residents’ chances to reach affiliation in their new living surroundings, and thus adjust to that social environment	15 older adults	≥65	Sheltered home	Not specified	Ethnographic approach	Thematic interviews; participant observation method	Detachment inside the assisted living facility Separateness from the social world outside the assisted living facility
Wan [45]	China	To gain a comprehensive understanding of the real challenges of social isolation faced by institutionalized older adults	6 older adults experiencing both objective and subjective social isolation	74-84	Nursing institution	≥6 months	Mixed methodological research	Semistructured interviews; participant observation method	Objective social isolation Subjective social isolation
Wei [46]	China	To conduct a thorough analysis of the causes and factors influencing social isolation among older people with diabetes	11 older people with diabetes who experience social isolation	60-84	Hospital	Not specified	Mixed methodological research	Semistructured interview	Changes in physical function Insufficient inner strength Weakening of social support Ineffective social coping
Zhang [47]	China	To understand the levels of interpersonal alienation among semidisabled older people in care institutions and to explore the underlying causes of high interpersonal alienation	10 semidisabled older adults with a score exceeding 2.8 on the General Social Alienation Scale	79-85	Nursing institution	≥6 months	Mixed methodological research	Semistructured interviews; participant observation method	Manifestations of interpersonal alienation among institutionalized older Adults with partial disabilities Causes of interpersonal alienation among institutionalized older adults with partial disabilities
Zhu [48]	China	To understand the manifestations and causes of social interaction barriers among partially disabled older adults in nursing homes	32 partially disabled older adults	≥65	Nursing institution	≥3 months	Mixed methodological research	Unstructured interview; participant observation method	Manifestations of social interaction barriers among institutionalized older adults with partial disabilities Causes of social interaction barriers among institutionalized older adults with partial disabilities

### Quality Assessment

According to the JBI criteria, all studies, except for 1, described at least 7 of the key items. All studies followed strict qualitative research methodologies and designs. Except for 4 studies, the remaining studies provided detailed statements of participants’ results. In total, 5 studies did not explicitly report whether appropriate institutional ethics approval had been obtained. Only 3 studies addressed the influence of the researcher on the research or the impact of the research on the researcher (Table 2), indicating a potential bias in the findings.

**Table 2:** Joanna Briggs Institute critical appraisal of included studies^a^.

Authors	1	2	3	4	5	6	7	8	9	10	Quality rating
Bartlett [35]	Yes	Yes	Yes	Yes	Yes	Yes	No	Yes	Yes	Yes	B
Buckley and McCarthy [36]	Yes	Yes	Yes	Yes	Yes	No	No	Yes	Yes	Yes	B
Cook et al [37]	Yes	Yes	Yes	Yes	Yes	No	No	No	Yes	Yes	B
Goll et al [38]	Yes	Yes	Yes	Yes	Yes	Yes	Yes	No	Yes	Yes	B
Li and Bai [39]	Yes	Yes	Yes	Yes	Yes	No	No	No	Unclear	Yes	B
Li [40]	Yes	Yes	Yes	Yes	Yes	No	No	Yes	Unclear	Yes	B
Mu and Yin [41]	Yes	Yes	Yes	Yes	Yes	No	No	Yes	Unclear	Yes	B
Neves et al [42]	Yes	Yes	Yes	Yes	Yes	Yes	No	Yes	Yes	Yes	B
Nielson et al [43]	Yes	Yes	Yes	Yes	Yes	Yes	Yes	Yes	Yes	Yes	A
Pirhonen et al [44]	Yes	Yes	Yes	Yes	Yes	Yes	Yes	Yes	Yes	Yes	A
Wan [45]	Yes	Yes	Yes	Yes	Yes	No	No	Yes	Unclear	Yes	B
Wei [46]	Yes	Yes	Yes	Yes	Yes	Yes	No	Yes	Yes	Yes	B
Zhang [47]	Yes	Yes	Yes	Yes	Yes	No	No	Yes	Unclear	Yes	B
Zhu [48]	Yes	Yes	Yes	Yes	Yes	No	No	No	Yes	Yes	B

^a^Domains: (1) Is there congruity between the stated philosophical perspective and the research methodology? (2) Is there congruity between the research methodology and the research question or objectives? (3) Is there congruity between the research methodology and the methods used to collect data? (4) Is there congruity between the research methodology and the representation and analysis of data? (5) Is there congruity between the research methodology and the interpretation of results? (6) Is there a statement locating the researcher culturally or theoretically? (7) Is the influence of the researcher on the research, and vice versa, addressed? (8) Are participants, and their voices, adequately represented? (9) Is the research ethical according to current criteria or, for recent studies, is there evidence of ethics approval by an appropriate body? (10) Do the conclusions drawn in the research report flow from the analysis, or interpretation, of the data?

### Main Findings of the Meta-Synthesis

#### Overview

This study generated 4 main themes (roots, manifestations, emotional experiences, and coping strategies of social isolation among older adults) and 13 subthemes.

**Figure 2:**
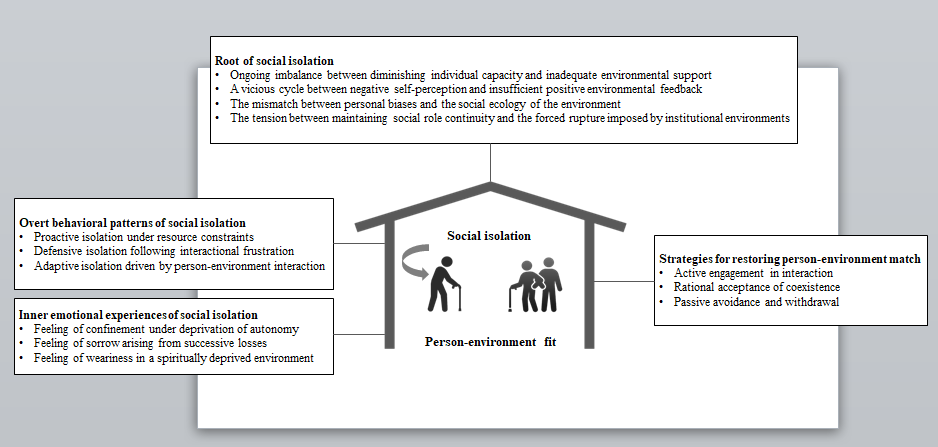
Thematic overview diagram.

The research team and qualified interpreters reviewed the identified themes to ensure alignment with the original authors’ intended interpretations, thereby enhancing the accuracy of thematic analysis across all included studies. These themes effectively reflect the social isolation experiences and perceptions of older people in LTCFs. Figure 2 shows the examination of each theme.

#### Theme 1: Person-Environment Mismatch as the Root of Social Isolation

##### Ongoing Imbalance Between Diminishing Individual Capacity and Inadequate Environmental Support

With advancing age, individuals experience a gradual decline in physiological functioning accompanied by a marked increase in the prevalence of chronic diseases. Older adults living in LTCFs frequently experience ongoing declines in mobility and capacity for independent daily living [49]. However, many LTC settings remain inadequate in providing supportive features—such as accessible infrastructure, mobility assistance, and functional spatial layout—thus failing to effectively offset the decline in residents’ functional capacity. This insufficiency of environmental support diminishes older adults’ autonomy and opportunities for external engagement, constraining their social participation and ultimately resulting in social isolation driven by the combined effects of functional decline and environmental misfit.

As I age and my health deteriorates, I need daily medication and injections. Over the past month, I have been receiving injections continuously and taking numerous pills each day. I truly have no desire to go out, nor is it physically feasible for me. Instead, I am just lying in bed

48

I used to be able to walk and visit friends, but now with this illness (diabetes) and my gout, along with foot sores, I can’t walk anymore. I can only sit or lie down, and I can’t go anywhere

46

##### A Vicious Cycle Between Negative Self-Perception and Insufficient Positive Environmental Feedback

Older adults living in institutional settings are susceptible to negative psychological states—including stigma, self-directed ageism, and feelings of guilt—triggered by aging and illness. In contrast, LTC environments generally provide far fewer affirming social interactions and psychological support than community contexts enriched by family and neighborhood ties, leaving older adults’ negative self-perceptions insufficiently mitigated or corrected. Over time, these enduring negative self-perceptions lead older adults to withdraw from social engagement due to fear of being judged or misunderstood, thereby further limiting their access to positive environmental experiences. As a result, a self-perpetuating vicious cycle develops, in which negative self-cognition and insufficient positive feedback from the environment continuously reinforce each other.

If you go out by [accessible taxi] ... you wonder if they’re gonna turn up

38

With this illness (diabetes), my savings and pension have been spent entirely on medication. Even going out to socialize incurs costs. Sometimes my children help out a little, but I feel embarrassed to ask for support; I don’t want to be a burden to them

46

##### The Mismatch Between Personal Biases and the Social Ecology of the Environment

After entering LTCFs, older adults often form social circles based on individual differences such as personal values, behavioral patterns, and past experiences. For example, some may instinctively distance themselves from peers with highly stigmatized illnesses and choose to interact only with those who are moderately impaired or still self-sufficient. Institutional environments often fail to provide diverse and flexible social groups; thus, the avoidance strategies individuals adopt to maintain self-identity become barriers to rebuilding their social networks.

These severely disabled older people individuals should not have been placed on our floor. They are entirely dependent on the caregivers for mobility, and their temperaments are often volatile, leaving the area unclean. We do not want to live on the same floor with them

39

I don’t want to go out. Those people lack manners. I went to college and used to be a Chinese teacher, so I can’t communicate clearly with them. Take my neighbor, for example—she’s practically crazy. She insults others and calls me names, saying I’m an introverted bore. She mocks my education, but her cultural level is far below mine. I refuse to share a room with her or interact with them

47

people don’t want to socialise with frail people

43

##### The Tension Between Maintaining Social Role Continuity and the Forced Rupture Imposed by Institutional Environments

Moving into an institutional setting imposes an abrupt and involuntary disruption on older adults’ established social functions and living contexts. Yet, the enclosed structure of LTC settings, the task-driven nature of care practices (focused on physical tasks rather than emotional connection), and the rigidity of institutional management collectively hinder the smooth transition and continuity of older adults’ social roles. These conditions expedite the erosion of their social identity and emotional support, ultimately exacerbating social isolation.

There’s nobody to talk to (...) no normal people, just the tea lady and the kitchen staff

47

I’ve been thinking about my friend who lives near Cuihu, but now that I’m living in this facility, I can’t go out. Oh well, I’m fine here (telling myself that). It’s probably best not to meet up anyway

47

I used to want to chat, so I would wait for the caregiver to come at mealtime just to say a few words. But she was always busy, rushing to feed me and then move on to the others. Gradually, I stopped trying to talk

40

#### Theme 2: Overt Behavioral Patterns of Social Isolation in the Context of Person-Environment Mismatch

##### Proactive Isolation Under Resource Constraints

Such isolation behaviors constitute a deliberate avoidance strategy adopted by older adults following an evaluation of their functional restrictions and the physical or supportive barriers within institutional environments [50]. Although these older adults often desire social participation, when they perceive that environmental supports (such as hearing aids, barrier-free facilities, or accessible activity spaces) are inadequate to compensate for physical deterioration, they reasonably foresee possible frustration or risk during social engagement. Consequently, in order to prevent possible communication barriers, physical strain, or unsafe conditions, they opt to limit or withdraw from social engagement. This behavior reflects a rational, cost-benefit–driven decision aimed at minimizing the adverse outcomes that may result from social interaction.

I do want to talk to someone, but my health and other conditions don’t allow it. Sometimes my spouse takes me out on a tricycle, but it still feels difficult to make friends or have a conversation

46

It’s not that I don’t want to go out and talk to people—it’s just that I’m too deaf. Even if someone tries to speak to me, they’d have to shout, and I still might not catch what they’re saying. So, I just stay in my room by myself

40

##### Defensive Isolation Following Interactional Frustration

This pattern of behavior arises from repeated negative interpersonal interactions experienced in institutional contexts. When older adults face frequent rejection or misunderstanding during social interactions, they are likely to develop a consistent pattern of negative attribution, placing the cause on others or on an unsupportive environment. As a defensive strategy, individuals deliberately retreat from social settings to shield themselves from additional interpersonal hurt and emotional pain. While this behavior offers short-term self-protection, in the long run, it depletes social capital and entrenches the individual’s isolation.

Ask for help and you’re turned down. That hurts ... It’s not worth the aggravation ... I don’t want to ask anybody for anything, nothing ... I don’t want another knock-back

38

And they don’t speak to me and I don’t speak to them. We are like bits of kids, oh dear God. To think that I have come to this

37

##### Adaptive Isolation Driven by Person-Environment Interaction

Certain older adults demonstrate reduced intrinsic motivation for social interaction, influenced by personality characteristics or life stage factors. At the same time, the social activities offered by institutional environments tend to be limited in variety, depth, and attractiveness, thus insufficient to foster residents’ motivation to engage. In this context, a passive equilibrium emerges between the individual’s limited social needs and the environment’s equally limited social offerings. Older adults’ seeming acceptance of solitude is not only a manifestation of personal preference but also a consequence of the environment’s insufficient provision of meaningful social engagement: “[Being alone] doesn’t bother me anymore” [38]*.*

#### Theme 3: Inner Emotional Experiences of Social Isolation in the Context of Person-Environment Mismatch

##### Feeling of Confinement Under Deprivation of Autonomy

The feeling of confinement arises from the tension between the individual’s desire for autonomy and the physical as well as administrative constraints of institutional settings. As older adults’ physical abilities deteriorate with age and illness, institutional environments often intensify restrictions through closed spatial layouts, rigid outing regulations, and standardized routines, resulting in a pronounced sense of being trapped within specific areas (eg, a room, a floor, or the entire facility). The confinement experience goes beyond physical restriction. It reflects a profound psychological response to the institutionalized deprivation of personal autonomy and indicates a severe loss of one’s sense of control over the environment.

I just feel cut off from the outside world that’s all ... I don’t feel I have contact with the outside world at all

36

you can get in alright but you can’t get out

35

##### Feeling of Sorrow Arising From Successive Losses

With the progression of aging and chronic illness, residents in LTCFs tend to endure a wider spectrum of losses than their peers, including diminished bodily autonomy, bereavement of close companions, separation from a spouse, and disruption of familiar environments. When faced with the loss of youth, vitality, and beloved relationships, certain older adults sink into a pervasive sorrow from ongoing losses, leading to an increasingly pessimistic outlook on life. During this journey marked by continual loss, some older people become absorbed in nostalgic recollections, rendering them more susceptible to distress and emotional isolation.

more of a torment I think ... That is the thing that gets me I start talking like this and then it goes out of my head. I have a hell of a job to get it back. It is awful really

35

All my family’s gone. I had to accept that. You see other people coming in with families. It does hurt. But you move on. You just move on, say, “Well, they’re lucky. Their family and that are still alive”

42

[My friend] who takes me shopping had been away, so I hadn’t been out for quite some time

38

##### Feeling of Weariness in a Spiritually Deprived Environment

Some older persons in LTCFs find life tedious and uninspiring. On the one hand, their daily routines are dictated by established care processes, leaving little possibility for autonomy or personal choice, resulting in a lack of novelty and variation in their lives. On the other hand, the restricted range of activities available in LTCFs frequently fails to meet their different interests, leading people to believe that life is dull and lifeless. This lack of mental stimulation can quickly lead to boredom, exacerbating their feelings of loneliness and isolation. The absence of cognitive stimulation can readily induce fatigue and exacerbate their social isolation.

There’s really nothing going on; I just can’t do anything all day, and I feel a sense of emptiness inside

48

life is people; we need to belong; we need to talk, we old lonely men (...) we see each other every day, but we don’t talk, we don’t even know what to do

42

#### Theme 4: Strategies for Restoring Person-Environment Match to Alleviate Social Isolation

##### Active Engagement in Interaction

Some older people deal with social isolation by actively engaging in social activities and socializing with others. By expanding their exposure to the outside world, such as participating in group activities or attending institutions for older adults, they might form new relationships or pursue personal hobbies, re-establish social links with the outside world, and gain a sense of belonging and importance.

The nursing home has provided us with a sound system specifically for square dancing after dinner. Essentially, as long as one can walk, everyone comes together daily to enjoy dancing

41

if you don’t participate you can be left out and feel isolated, so I participate in some of the things

42

It’s nice to sit with the nurses and talk and laugh about things with them when they are not busy working

44

##### Rational Acceptance of Coexistence

Another group of older people copes by rationally accepting the institutional context and adjusting their mindsets to achieve inner balance, a strategy closely tied to the LTCF-specific experience of “relocating from family to institutional care.” Over time, they seek inner peace by changing their mindset and progressively adjusting to live in harmony with their caregivers and cohabitants. This logical acceptance keeps individuals from experiencing more psychological distress, even though they still feel alienated from the outside world.

Now that the children have to work and take care of their own kids, they are under a lot of pressure and don’t have time to look after us. That’s why we’ve come here. We have to adapt to this environment, take care of ourselves, and avoid getting sick so we don’t add to our children’s burdens

39

Good child, I’m old now, and I’m stuck here. My spouse passed away early. My health isn’t good; I have high blood pressure and have been on medication for a long time. I just don’ have the energy to be around those lively people. Right now, I’m just taking it one day at a time and not thinking too much about it

48

##### Passive Avoidance and Withdrawal

Some older people adopt passive coping behaviors in response to LTCF-related social barriers, forming a maladaptive cycle of isolation. This coping strategy involves passive avoidance and withdrawal from social interactions among older people. Due to the fixed, inflexible schedules and routines in LTCF, residents’ personalized social needs often cannot be met. In response, some older people choose to withdraw from institutional social activities, avoid social environments, and reduce interaction with others as a form of passive coping. Long-term avoidance may harm their psychological and physical health, making them feel even more alone. Over time, this creates a vicious cycle, in which older people retreat for extended periods of time, no longer seeking opportunities to contact with the outside world, and may even acquire suicidal ideation.

Well I just can’t be bothered ... I just don’t want to make the effort. I mean, I don’t get up very early in the mornings ... When I feel lonely I don’t want to do anything ... [I’ve] just lost interest

38

## Discussion

### Principal Findings

This synthesis identifies person-environment mismatch (encompassing 4 interrelated dimensions: functional decline vs insufficient environmental support, negative self-perception vs feedback deficit, personal bias vs homogeneous institutional ecology, and role disruption vs rigid management) as the core mechanism driving social isolation of older people in LTCFs. This mismatch manifests in 3 behavioral patterns (proactive, defensive, and adaptive isolation) and 3 emotional states (confinement, sorrow, and weariness), with residents adopting active engagement, rational acceptance, or passive withdrawal as coping strategies. Notably, LTCF residents’ social isolation is a unique, institution-specific phenomenon (not an extension of community-dwelling), and interventions should prioritize restoring person-environment congruence—providing targeted guidance for optimizing institutional care, strengthening social support, and fostering active social participation among older people in LTCFs.

### Core Mechanism Elaboration: Person-Environment Mismatch as the Root Cause of Social Isolation Among Older People in LTCFs

This study confirms that person-environment mismatch is the core driver of social isolation among older adults in LTCFs, validating the P-E fit theory and extending its utility to institutional older people care via empirical evidence. These 4 interrelated dimensions of this mismatch collectively contribute to social isolation, with synergistic rather than independent effects. First, the gap between declining physical capacity (eg, mobility loss from diabetes or gout) and insufficient environmental support (eg, lack of barrier-free facilities) forms a “functional restriction→participation barrier” loop. This finding is consistent with previous research linking environmental inadequacy to social withdrawal [51] and further highlights that this issue is persistent in LTCFs, where impaired mobility often confines residents to their beds. Second, negative self-perceptions—fueled by financial strain (eg, pensions spent on medication) or distrust in external support (eg, worries about accessible taxis)—are not mitigated by LTCFs’ weak emotional feedback. Unlike community settings with family ties, this gap exacerbates self-directed ageism and active avoidance [46], and this study adds that such cycles are more intractable in closed institutions. Third, personal biases (based on education or health status) collide with LTCFs’ homogeneous social ecology. While Zhang et al [52] noted institutional homogeneity limits adaptation, this study further finds that the absence of flexible social groupings turns preferences (eg, avoiding severely disabled peers) into long-term isolation. Fourth, closed management and task-oriented care disrupt social roles (eg, losing “friend” or “teacher” identities), eroding emotional anchors. Consistent with the findings of Huang et al [53] on role disruption and isolation, this study highlights that LTCFs’ lack of role-transition support accelerates this erosion. Together, these dimensions reveal that person-environment mismatch in LTCFs is a cumulative, interactive process—each dimension reinforces the others—making isolation more deeply rooted than in community or home settings.

### Analysis of 3D Environmental Conflicts: Reflections on Person-Environment Mismatch

#### Overview

Drawing on the study’s empirical findings, environmental conflicts in LTCFs can be categorized into 3 dimensions: physical, social, and psychological. Each dimension reflects specific manifestations of person-environment mismatch and deepens social isolation, consistent with the multidimensional environmental framework of Smith et al [54]. At the same time, this study found that the social isolation performance of older persons in LTCFs could be classified as “proactive,” “defensive,” and “adaptive,” which is consistent with previous research findings [46,52].

#### Physical Environment Versus Physiological Needs: Aggravating “Sense of Confinement” and “Proactive Isolation”

Conflicts here stem from LTCFs’ failure to align spatial-temporal arrangements with the declining physical capacity of older people. First, rigid time management (eg, unified meal or rest times) creates “flattened time,” contradicting residents’ need for autonomy. This exacerbates meaninglessness and passive isolation [51], and our results add that such rigidity leaves no room for adapting to individual energy levels (eg, residents too fatigued from medication to participate in scheduled activities). Second, insufficient barrier-free facilities (eg, lack of walking aids) trap mobility-impaired residents—our cases show that those with foot injuries or gout cannot reach activity areas, reinforcing physical confinement [52]. Third, closed spatial layouts sever external connections; residents unable to meet friends outside (as in our results) rely solely on internal resources, and when these are scarce, isolation deepens.

#### Social Environment Versus Social Needs: Triggering “Loneliness” and “Defensive Isolation”

This dimension’s core issue is the mismatch between homogeneous social services and diverse individual needs. First, unified care (eg, passive waiting for meals or bathing) ignores social preferences—extroverts need group interactions, while introverts need one-on-one chats. We have a former Chinese teacher who is unable to communicate with peers with lower cultural levels, and this case illustrates this point [47]; this mismatch increases withdrawal. Second, caregivers prioritize tasks over emotional support; our results show that residents wanting to chat during meals are rushed, leading to “defensive isolation” (eg, no longer initiating interactions) [53-56]. Third, tense peer relationships—driven by health discrimination (eg, avoiding severely disabled peers)—undermine belonging. Our cases of residents refusing roommates who insult them reflect this, aligning with the observation of Nielson et al [43] of peer exclusion worsening isolation. Additionally, this study also believes that some older people may find it difficult to express their feelings of social isolation. This can make it difficult for caregivers to understand and sympathize with older people because of limitations and a lack of knowledge about the level of expertise of caregivers.

#### Psychological Environment Versus Psychological Resilience: Strengthening “Sense of Powerlessness” and “Adaptive Isolation”

Imbalances here lie in LTCFs’ failure to support older people’s mental coping with isolation. First, caregivers lack psychological skills to recognize loneliness or anxiety; uniformity of feeling among people remains impossible, and this study summarizes these themes as “active participation in interactions,” “rational acceptance of coexistence,” and “passive avoidance and withdrawal.” Some older people stop expressing feelings when unheard, fostering powerlessness [57,58]. Second, monotonous activities (eg, repetitive handicrafts) fail to meet self-worth needs; older people in previous research have described “empty lives,” leading to “adaptive isolation” (accepting loneliness) [59-62]. Third, static assessments ignore changing needs of older people (eg, hearing loss requiring hearing aids leads to a sense of powerlessness in expressing needs), and this static care deepens passive withdrawal.

### Practical Implications: Person-Environment Matching-Based Interventions for Social Isolation

#### Overview

Guided by the study’s core findings—effective coping strategies (eg, active participation and rational acceptance) and 3D environmental conflicts—interventions should focus on “repairing person-environment mismatch.” Targeted, actionable measures are proposed across physical, social, and psychological dimensions to facilitate older adults’ transition from “passive restriction” to “active adaptation,” aligning with P-E fit theory’s intervention orientation [23].

#### Optimizing the Physical Environment: Enhancing “Adaptability” to Reduce Physical Barriers to Socialization

##### Implement Flexible Time Management

Referring to older adults’ need to “arrange activities independently” in the results, LTCFs should allow older adults to choose meal and rest times within a certain range (eg, “providing 2 meal periods in the morning and evening”) [44]. Posters listing daily flexible slots should be displayed in residential wings. LTCFs could increase participation in 2 or more weekly timed activities (eg, 15:00 calligraphy and 17:30 group walks). This reduces the sense of meaninglessness caused by “flattened time”—consistent with the “temporal autonomy” intervention [63].

##### Construct Semiopen Spatial Layouts

Within closed management, facilities can establish weekly outing schedules (eg, community park visits on Wednesday mornings and friend visits on Friday afternoons), with 1 staff member assigned to every 6 residents. On a monthly basis, facilities can invite 2 to 3 community volunteers (eg, retired teachers and craftsmen) to hold on-site interactive activities such as paper-cutting workshops [64]. These practices help older people reconnect with the outside world and reduce their sense of isolation from external society.

#### Reconstructing the Social Environment: Strengthening “Supportiveness” to Improve the Quality of Social Interaction

##### Transform Caregiver Role Positioning

LTCFs should strengthen training for caregivers in psychological support skills, transforming them from “task executors” to “social facilitators.” For example, caregivers should “take the initiative to chat with older people during meals and listen to their feelings,” integrating social support for older people into daily nursing work: nursing staff actively care about the older people’s mood and feelings during meal delivery or daily patrols, communicate for about 5 minutes each time, and record their social and reaction situations in nursing records. This reduces the occurrence of “defensive isolation”—aligning with the view of Caspar and O’Rourke [65].

##### Foster an Inclusive Peer Atmosphere

Through “collective ice-breaking activities” and “peer mutual aid programs” [66] (eg, “pairing 1 high-functioning resident with 1 severely disabled resident for 30-minute daily interactions”), it breaks the prejudice of “unwillingness to live with severely disabled peers” and reconstructs positive peer relationships and eliminates “health discrimination.”

#### Improving the Psychological Environment: Enhancing “Empowerment” to Boost Individual Coping Resilience

##### Strengthen Collaborative Support Between Families and LTCFs

Facilities can organize monthly “family activity days” (eg, parent-child tea parties on the second Sunday of each month) and provide free video call stations [67]. Staff can assist residents who are not familiar with digital devices, and each resident can make three 15-minute video calls per week. In addition, facilities can share “resident social progress reports” with family members quarterly to encourage family involvement in social goal-setting.

##### Establish a Dynamic Psychological Assessment Mechanism

LTCFs should regularly assess older adults’ psychological states (eg, “loneliness level, social willingness”) and changing needs (eg, “hearing loss requiring hearing aids”). Timely adjustments to intervention strategies can prevent “passive withdrawal due to unrecognized needs”—consistent with the proposal of Davies et al [68] for “dynamic monitoring to match psychological needs.”

To ensure implementation, establish a three-tier oversight system: (1) micro: caregivers track daily intervention adherence via logs, (2) meso: LTCF managers conduct monthly spot checks (eg, verifying flexible meal times), and (3) macro: collaborate with local aging agencies to audit quarterly and provide funding for barrier-free renovations. This system ensures that interventions translate from policy to practice, driving residents’ shift to active adaptation.

### Strengths and Limitations

The strengths of this qualitative systematic study and meta-synthesis include a good grasp of older people’s social isolation experiences. All relevant studies were identified using a careful search approach. However, unavoidable heterogeneity exists due to cross-national variations in LTCF resident characteristics and the care services provided. Factors including the sex and health status of the study population, the quality of care provided by caregivers, and the care environment quality may all influence how older people perceive their living environment. A key limitation is the uneven sex distribution among participants in the included studies, with most reporting a higher proportion of the female population. This sex imbalance may have influenced the salience of specific thematic findings. Future qualitative studies should aim for more balanced sex recruitment to capture sex-specific experiences of social isolation. At the same time, it is important to note that although many of the included studies were conducted from a Chinese perspective, several others originated from different countries. Cultural variations in older adults’ perceptions of social isolation may additionally introduce bias into the interpretation of study findings.

### Conclusions

This qualitative meta-synthesis explores social isolation experiences among older people in LTCFs and analyzes the underlying causes, behavioral manifestations, emotional experiences, and coping strategies associated with this phenomenon. Although this study describes social isolation experiences across both agentic and structural dimensions, most participants perceived social isolation as an individual challenge shaped by the interaction between their internal perceptions and the external institutional environment. As such, older people had to deal with these experiences using the social and individual strategies they had developed [69].

Additionally, there is a need to continue exploring the role of caregivers and other aged care staff in the prevention and management of social isolation of older people in LTCFs. Focusing on the needs of older people in LTCFs, through environmental transformation, relationship reconstruction, and dignity empowerment, the symbiotic relationship between humans and the environment can be reconstructed. Future research should further explore the quantification of person-environment matching in LTCFs—including the development of valid assessment tools—to enable more targeted interventions for social isolation.

## Appendix

Multimedia Appendix 1 PRISMA checklist.

Multimedia Appendix 2 ENTREQ reporting guidelines.

Multimedia Appendix 3 Literature search strategy and search terms.

## Abbreviations

ENTREQ: Enhancing Transparency in Reporting the Synthesis of Qualitative Research
JBI: Joanna Briggs Institute
LTC: long-term care
LTCF: long-term care facility
P-E fit: person-environment fit
PICOS: population, intervention, context, outcome, study design
PRISMA: Preferred Reporting Items for Systematic Reviews and Meta-Analyses

## Data Availability

The datasets generated and/or analyzed during this study are available from the corresponding author upon reasonable request.
