# Supplementary material for: “Living Inside the Walls”: Systematic Review and Qualitative Meta-Synthesis
Source: JMIR Aging. 2026 Apr 17;9:e78142. doi: 10.2196/78142 (PMC13135159; doi:10.2196/78142)
Supplement: Multimedia Appendix 2 [file aging_v9i1e78142_app2.docx]

**Enhancing transparency in reporting the synthesis of qualitative research: the ENTREQ statement**

| **No** | **Item** | **Guide and description** | **Location where item is reported** |
| --- | --- | --- | --- |
| 1 | Aim | State the research question the synthesis addresses. | **PAGE 6** |
| 2 | Synthesis  methodology | Identify the synthesis methodology or theoretical framework which underpins the synthesis, and describe the rationale for choice of methodology (e.g. meta-ethnography, thematic synthesis, critical interpretive synthesis, grounded theory synthesis realist synthesis, meta-aggregation, meta-study, framework synthesis) | **PAGE 6** |
| 3 | Approach to  searching | Indicate whether the search was pre-planned (comprehensive search strategies to seek all available studies) or iterative (to  seek all available concepts until they theoretical saturation is achieved). | **PAGE 6-7** |
| 4 | Inclusion criteria | Specify the inclusion/exclusion criteria (e.g. in terms of population, language, year limits, type of publication study type). | **PAGE 7** |
| 5 | Data sources | Describe the information sources used (e.g.electronic databases (MEDLINE, EMBASE, CINAHL, psycINFO, Econlit), grey  literature databases (digital thesis,policy reports), relevant organizational websites, experts, information specialists,generic web searches (Google Scholar) hand searching, reference lists) and when the searches conducted; provide the rationale for using the data sources. | **PAGE 7** |
| 6 | Electronic Search  strategy | Describe the literature search (e.g. provide electronic search strategies with population terms,clinical or health topic terms,experiential or social phenomena related terms, filters for qualitative research, and search limits) | **Supplementary File 2** |
| 7 | Study screening  methods | Describe the process of study screening and sifting (e.g. title, abstract and full test review, number of independent reviewers who screened studies) | **PAGE 8** |
| 8 | Study characteristics | Present the characteristics of the included studies (e.g. year of publication, country, population, number of participants, data collection, methodology, analysis, research questions). | **TABLE 1** |
| **No** | **Item** | **Guide and description** | **Location where item is reported** |
| 9 | Study selection  results | Identify the number of studies screened and provide reasons for study exclusion (e.g. for comprehensive searching, provide numbers of studies screened and reasons for exclusion indicated in a figure/flowchart; for iterative searching describe reasons for study exclusion and inclusion based on modifications r the research question and/or contribution to theory development). | **PAGE 8-9** |
| 10 | Rationale for  appraisal | Describe the rationale and approach used to appraise the included studies or selected findings (e.g. assessment of conduct (validity and robustness), assessment of reporting (transparency), assessment of content and utility of the findings). | **PAGE 10** |
| 11 | Appraisal items | State the tools, frameworks and criteria used to appraise the studies or selected findings (e.g. Existing toolk: CASP, QARLCOREQ, Mays and Pope [25]: reviewer developed tools; describe the domains assessed: research team, study design, data analysis and interpretations, reporting). | **PAGE 17** |
| 12 | Appraisal process | Indicate whether the appraisal was conducted independently by more than one reviewer and if consensus was required. | **PAGE 10** |
| 13 | Appraisal results | Present results of the quality assessment and indicate which articles, if any, were weighted/excluded based on the  assessment and give the rationale. | **Table 17** |
| 14 | Data extraction | Indicate which sections of the primary studies were analyzed and how were the data extracted from the primary studies? (e.g. all text under the headings 'results /conclusions"were extracted electronically and entered into a computer software). | **PAGE 10-11** |
| 15 | Software | State the computer software used, if any. | **Data management and deduplication with EndNote 20.** |
| 16 | Number of  reviewers | Identify who was involved in coding and analysis. | **PAGE 10** |
| 17 | Coding | Describe the process for coding of data (e.g. line by line coding to search for concepts). | **PAGE 10-11** |
| 18 | Study comparison | Describe how were comparisons made within and across studies (e.g. subsequent studies were coded into pre-existing concepts,and new concepts were created when deemed necessary) . | **PAGE 10-11** |
| **No** | **Item** | **Guide and description** | **Location where item is reported** |
| 19 | Derivation of  themes | Explain whether the process of deriving the themes or constructs was inductive or deductive. | **PAGE 10-11** |
| 20 | Quotations | Provide quotations from the primary studies to illustrate themes/constructs, and identify whether the quotations were participant quotations of the author's interpretation. | **PAGE 18-25** |
| 21 | Synthesis output | Present rich, compelling and useful results that go beyond a summary of the primary studies (e.g. new interpretation,  models of evidence, conceptual models, analytical framework, development of a new theory or construct) | **PAGE 18** |
