# Supplementary material for: “Living Inside the Walls”: Systematic Review and Qualitative Meta-Synthesis
Source: JMIR Aging. 2026 Apr 17;9:e78142. doi: 10.2196/78142 (PMC13135159; doi:10.2196/78142)
Supplement: Multimedia Appendix 3 [file aging_v9i1e78142_app3.docx]

| **Database** | **Result** | **Search String** |
| --- | --- | --- |
| Cochrane library | 64 | **#1** [mh "Social Isolation"]  **#2** (social isolation OR social exclusion OR social alienation):ti,ab,kw  **#3 #1 OR #2**  **#4** [mh Aged]  **#5** (aged OR elder* OR old OR old age* OR aging* OR aged* OR geriatric* OR senior* OR older*):ti,ab,kw  **#6 #4 OR #5**  **#7** [mh "Nursing Homes"] OR [mh "Long-Term Care"] OR [mh "Residential Facilities"] OR [mh "Home Care Services"] OR [mh "Homes for the Aged"] OR [mh "Home Care Agencies"] OR [mh "Home Health Nursing"] OR [mh "Family Nursing"] OR [mh "Rehabilitation Centers"]  **#8** ("nursing homes" OR "home care" OR "extended care" OR "longterm care" OR "long-term care" OR "residential facilities" OR "home care services" OR "homes for the aged" OR "home care agencies" OR "home health nursing" OR "family nursing" OR "homecare" OR "aged care" OR "convalescent home" OR "rehabilitation centers" OR "elderly care facility" OR "elderly care" OR "long term nursing"):ti,ab,kw  **#9 #7 OR #8**  **#10** ("Experience" OR "Perception" OR "feelings" OR "Perspectives" OR "Opinions" OR "Attitude" OR "Views" OR "Cognition" OR "need" OR "Expectation" OR "Emotions" OR "Empiricism" OR "demands" OR "Attitude" OR "Interviews" OR "Motivation" OR "Cognition" OR "qualitative research" OR "Phenomenology" OR "grounded theory" OR "case study" OR "Ethnography" OR "action research" OR "thematic analysis" OR "content analysis" OR "focus groups" OR "interviews as topic" OR "case study" OR "anthropology cultural" OR "health services research" OR "framework analysis"):ti,ab,kw  **#11 #3 AND #6 AND #9 AND #10** |
| CIINAHL (EBSCOhost) | 88 | **#1** TI ( social isolation OR social exclusion OR social alienation ) OR AB ( social isolation OR social exclusion OR social alienation )  **#2** TI ( aged OR elder* OR old OR old age* OR aging* OR aged* OR geriatric* OR senior* OR older* ) OR AB ( aged OR elder* OR old OR old age* OR aging* OR aged* OR geriatric* OR senior* OR older* )  **#3** TI ( "nursing homes" OR "home care" OR "extended care" OR "longterm care" OR "long-term care" OR "residential facilities" OR "home care services" OR "homes for the aged" OR "home care agencies" OR "home health nursing" OR "family nursing" OR "homecare" OR "aged care" OR "convalescent home" OR "rehabilitation centers" OR "elderly care facility" OR "elderly care" OR "long term nursing" ) OR AB ( "nursing homes" OR "home care" OR "extended care" OR "longterm care" OR "long-term care" OR "residential facilities" OR "home care services" OR "homes for the aged" OR "home care agencies" OR "home health nursing" OR "family nursing" OR "homecare" OR "aged care" OR "convalescent home" OR "rehabilitation centers" OR "elderly care facility" OR "elderly care" OR "long term nursing" )  **#4** TI ( "Experience" OR "Perception" OR "feelings" OR "Perspectives" OR "Opinions" OR "Attitude" OR "Views" OR "Cognition" OR "need" OR "Expectation" OR "Emotions" OR "Empiricism" OR "demands" OR "Attitude" OR "Interviews" OR "Motivation" OR "Cognition" OR "qualitative research" OR "Phenomenology" OR "grounded theory" OR "case study" OR "Ethnography" OR "action research" OR "thematic analysis" OR "content analysis" OR "focus groups" OR "interviews as topic" OR "case study" OR "anthropology cultural" OR "health services research" OR "framework analysis" ) OR AB ( "Experience" OR "Perception" OR "feelings" OR "Perspectives" OR "Opinions" OR "Attitude" OR "Views" OR "Cognition" OR "need" OR "Expectation" OR "Emotions" OR "Empiricism" OR "demands" OR "Attitude" OR "Interviews" OR "Motivation" OR "Cognition" OR "qualitative research" OR "Phenomenology" OR "grounded theory" OR "case study" OR "Ethnography" OR "action research" OR "thematic analysis" OR "content analysis" OR "focus groups" OR "interviews as topic" OR "case study" OR "anthropology cultural" OR "health services research" OR "framework analysis" )  **#5 #1 AND #2 AND #3 AND #4** |
| Embase | 806 | **#1** 'social isolation'/exp  **#2** 'social isolation':ab,ti OR 'social exclusion':ab,ti OR 'social alienation':ab,ti  **#3 #1 OR #2**  **#4** 'aged'/exp  **#5** aged:ab,ti OR elder*:ab,ti OR old:ab,ti OR ’old age*’:ab,ti OR aging*:ab,ti OR aged*:ab,ti OR geriatric*:ab,ti OR senior*:ab,ti OR older*:ab,ti  **#6 #4 OR #5**  **#7** 'nursing homes'/exp OR 'long-term care'/exp OR 'residential facilities'/exp OR 'home care services'/exp OR 'homes for the aged'/exp OR 'home care agencies'/exp OR 'home health nursing'/exp OR 'family nursing'/exp OR 'rehabilitation centers'/exp  **#8** 'nursing homes':ab,ti OR 'home care':ab,ti OR 'extended care':ab,ti OR 'longterm care':ab,ti OR 'long-term care':ab,ti OR 'residential facilities':ab,ti OR 'home care services':ab,ti OR 'homes for the aged':ab,ti OR 'home care agencies':ab,ti OR 'home health nursing':ab,ti OR 'family nursing':ab,ti OR homecare:ab,ti OR 'aged care':ab,ti OR 'convalescent home':ab,ti OR 'rehabilitation centers':ab,ti OR 'elderly care facility':ab,ti OR 'elderly care':ab,ti OR 'long term nursing':ab,ti  **#9 #7 OR #8**  **#10** experience:ab,ti OR perception:ab,ti OR feelings:ab,ti OR perspectives:ab,ti OR opinions:ab,ti OR views:ab,ti OR need:ab,ti OR expectation:ab,ti OR emotions:ab,ti OR empiricism:ab,ti OR demands:ab,ti OR attitude:ab,ti OR interviews:ab,ti OR motivation:ab,ti OR cognition:ab,ti OR 'qualitative research':ab,ti OR phenomenology:ab,ti OR 'grounded theory':ab,ti OR ethnography:ab,ti OR 'action research':ab,ti OR 'thematic analysis':ab,ti OR 'content analysis':ab,ti OR 'focus groups':ab,ti OR 'interviews as topic':ab,ti OR 'case study':ab,ti OR 'anthropology cultural':ab,ti OR 'health services research':ab,ti OR 'framework analysis':ab,ti  **#11 #3 AND #6 AND #9 AND #10** |
| Psyinfo (EBSCOhost) | 137 | **#1** TI ( social isolation OR social exclusion OR social alienation ) OR AB ( social isolation OR social exclusion OR social alienation )  **#2** TI ( aged OR elder* OR old OR old age* OR aging* OR aged* OR geriatric* OR senior* OR older* ) OR AB ( aged OR elder* OR old OR old age* OR aging* OR aged* OR geriatric* OR senior* OR older* )  **#3** TI ( "nursing homes" OR "home care" OR "extended care" OR "longterm care" OR "long-term care" OR "residential facilities" OR "home care services" OR "homes for the aged" OR "home care agencies" OR "home health nursing" OR "family nursing" OR "homecare" OR "aged care" OR "convalescent home" OR "rehabilitation centers" OR "elderly care facility" OR "elderly care" OR "long term nursing" ) OR AB ( "nursing homes" OR "home care" OR "extended care" OR "longterm care" OR "long-term care" OR "residential facilities" OR "home care services" OR "homes for the aged" OR "home care agencies" OR "home health nursing" OR "family nursing" OR "homecare" OR "aged care" OR "convalescent home" OR "rehabilitation centers" OR "elderly care facility" OR "elderly care" OR "long term nursing" )  **#4** TI ( "Experience" OR "Perception" OR "feelings" OR "Perspectives" OR "Opinions" OR "Attitude" OR "Views" OR "Cognition" OR "need" OR "Expectation" OR "Emotions" OR "Empiricism" OR "demands" OR "Attitude" OR "Interviews" OR "Motivation" OR "Cognition" OR "qualitative research" OR "Phenomenology" OR "grounded theory" OR "case study" OR "Ethnography" OR "action research" OR "thematic analysis" OR "content analysis" OR "focus groups" OR "interviews as topic" OR "case study" OR "anthropology cultural" OR "health services research" OR "framework analysis" ) OR AB ( "Experience" OR "Perception" OR "feelings" OR "Perspectives" OR "Opinions" OR "Attitude" OR "Views" OR "Cognition" OR "need" OR "Expectation" OR "Emotions" OR "Empiricism" OR "demands" OR "Attitude" OR "Interviews" OR "Motivation" OR "Cognition" OR "qualitative research" OR "Phenomenology" OR "grounded theory" OR "case study" OR "Ethnography" OR "action research" OR "thematic analysis" OR "content analysis" OR "focus groups" OR "interviews as topic" OR "case study" OR "anthropology cultural" OR "health services research" OR "framework analysis" )  **#5 #1 AND #2 AND #3 AND #4** |
| PubMed | 596 | **#1** "social isolation"[MeSH Terms]  **#2** "social isolation"[Title/Abstract] OR "social exclusion"[Title/Abstract] OR "social alienation"[Title/Abstract]  **#3 #1 OR #2**  **#4** "aged"[MeSH Terms]  **#5** "aged"[Title/Abstract] OR "elder*"[Title/Abstract] OR "old"[Title/Abstract] OR "old age*"[Title/Abstract] OR "aging*"[Title/Abstract] OR "aged*"[Title/Abstract] OR "geriatric*"[Title/Abstract] OR "senior*"[Title/Abstract] OR "older*"[Title/Abstract]  **#6 #4 OR #5**  **#7** "nursing homes"[MeSH Terms] OR "long-term care"[MeSH Terms] OR "residential facilities"[MeSH Terms] OR "home care services"[MeSH Terms] OR "homes for the aged"[MeSH Terms] OR "home care agencies"[MeSH Terms] OR "home health nursing"[MeSH Terms] OR "family nursing"[MeSH Terms] OR "rehabilitation centers"[MeSH Terms]  **#8** "nursing homes"[Title/Abstract] OR "home care"[Title/Abstract] OR "extended care"[Title/Abstract] OR "longterm care"[Title/Abstract] OR "long-term care"[Title/Abstract] OR "residential facilities"[Title/Abstract] OR "home care services"[Title/Abstract] OR "homes for the aged"[Title/Abstract] OR "home care agencies"[Title/Abstract] OR "home health nursing"[Title/Abstract] OR "family nursing"[Title/Abstract] OR "homecare"[Title/Abstract] OR "aged care"[Title/Abstract] OR "convalescent home"[Title/Abstract] OR "rehabilitation centers"[Title/Abstract] OR "elderly care facility"[Title/Abstract] OR "elderly care"[Title/Abstract] OR "long term nursing"[Title/Abstract]  **#9 #7 OR #8**  **#10** "Experience"[Title/Abstract] OR "Perception"[Title/Abstract] OR "feelings"[Title/Abstract] OR "Perspectives"[Title/Abstract] OR "Opinions"[Title/Abstract] OR "Attitude"[Title/Abstract] OR "Views"[Title/Abstract] OR "Cognition"[Title/Abstract] OR "need*"[Title/Abstract] OR "Expectation"[Title/Abstract] OR "Emotions"[Title/Abstract] OR "Empiricism"[Title/Abstract] OR "demands"[Title/Abstract] OR "Attitude"[Title/Abstract] OR "Interviews"[Title/Abstract] OR "Motivation"[Title/Abstract] OR "Cognition"[Title/Abstract] OR "qualitative research"[Title/Abstract] OR "Phenomenology"[Title/Abstract] OR "grounded theory"[Title/Abstract] OR "case study"[Title/Abstract] OR "Ethnography"[Title/Abstract] OR "action research"[Title/Abstract] OR "thematic analysis"[Title/Abstract] OR "content analysis"[Title/Abstract] OR "focus groups"[Title/Abstract] OR "interviews as topic"[Title/Abstract] OR "case study"[Title/Abstract] OR "anthropology cultural"[Title/Abstract] OR "health services research"[Title/Abstract] OR "framework analysis"[Title/Abstract]  **#11 #3 AND #6 AND #9 AND #10** |
| Scopus | 1206 | **#1** TITLE-ABS-KEY ("social isolation " OR "social exclusion " OR "social alienation ")  **#2** TITLE-ABS-KEY ("aged" OR "elder*" OR "old" OR "old age*" OR "aging*" OR "aged*" OR "geriatric*" OR "senior*" OR "older*")  **#3** TITLE-ABS-KEY ("nursing homes" OR "home care" OR "extended care" OR "longterm care" OR "long-term care" OR "residential facilities" OR "home care services" OR "homes for the aged" OR "home care agencies" OR "home health nursing" OR "family nursing" OR "homecare" OR "aged care" OR "convalescent home" OR "rehabilitation centers" OR "elderly care facility" OR "elderly care" OR "long term nursing")  **#4** TITLE-ABS-KEY ("Experience" OR "Perception" OR "feelings" OR "Perspectives" OR "Opinions" OR "Attitude" OR "Views" OR "Cognition" OR "need" OR "Expectation" OR "Emotions" OR "Empiricism" OR "demands" OR "Attitude" OR "Interviews" OR "Motivation" OR "Cognition" OR "qualitative research" OR "Phenomenology" OR "grounded theory" OR "case study" OR "Ethnography" OR "action research" OR "thematic analysis" OR "content analysis" OR "focus groups" OR "interviews as topic" OR "case study" OR "anthropology cultural" OR "health services research" OR "framework analysis")  **#5 #1 AND #2 AND #3 AND #4** |
| Web of Science | 602 | **#1** TS=("social isolation" OR "social exclusion" OR "social alienation")  **#2** TS=("aged" OR "elder*" OR "old" OR "old age*" OR "aging*" OR "aged*" OR "geriatric*" OR "senior*" OR "older*")  **#3** TS=("nursing homes" OR "home care" OR "extended care" OR "longterm care" OR "long-term care" OR "residential facilities" OR "home care services" OR "homes for the aged" OR "home care agencies" OR "home health nursing" OR "family nursing" OR "homecare" OR "aged care" OR "convalescent home" OR "rehabilitation centers" OR "elderly care facility" OR "elderly care" OR "long term nursing")  **#4** TS=("Experience" OR "Perception" OR "feelings" OR "Perspectives" OR "Opinions" OR "Attitude" OR "Views" OR "Cognition" OR "need" OR "Expectation" OR "Emotions" OR "Empiricism" OR "demands" OR "Attitude" OR "Interviews" OR "Motivation" OR "Cognition" OR "qualitative research" OR "Phenomenology" OR "grounded theory" OR "case study" OR "Ethnography" OR "action research" OR "thematic analysis" OR "content analysis" OR "focus groups" OR "interviews as topic" OR "case study" OR "anthropology cultural" OR "health services research" OR "framework analysis")  **#5 #1 AND #2 AND #3 AND #4** |
| CNKI | 340 | **#1** 主题词："老年" + "老年人" + "老人"  **#2** 主题词："社会隔离" + "社会网络" + "社会孤立" + "社会疏离"  **#3** 主题词："长期照护" + "照护" + "长期护理" + "养老机构" + "疗养院" + "养老" + "养老服务" + "居家" + "医养结合"  **#4** 主题词："体验" + "看法" + "感受" + "观点" + "感知" + "经历" + "态度" + "认知" + "需求" + "经验" + "期望" + "质性研究" + "定性研究" + "现象学" + "扎根理论" + "案例研究" + "民族志" + "行动研究" + "诠释主义" + "建构主义" + "主题分析" + "内容分析" + "焦点小组" + "访谈"  **#5 #1 AND #2 AND #3 AND #4** |
| CBM | 30 | **#1** "老年"[常用字段:智能] OR "老年人"[常用字段:智能] OR "老人"[常用字段:智能]  **#2** "社会隔离"[常用字段:智能] OR "社会网络"[常用字段:智能] OR "社会孤立"[常用字段:智能] OR "社会疏离"[常用字段:智能]  **#3** "长期照护"[常用字段:智能] OR "照护"[常用字段:智能] OR "长期护理"[常用字段:智能] OR "养老机构"[常用字段:智能] OR "疗养院"[常用字段:智能] OR "养老"[常用字段:智能] OR "养老服务"[常用字段:智能] OR "居家"[常用字段:智能] OR "医养结合"[常用字段:智能]  **#4** "体验"[常用字段:智能] OR "看法"[常用字段:智能] OR "感受"[常用字段:智能] OR "观点"[常用字段:智能] OR "感知"[常用字段:智能] OR "经历"[常用字段:智能] OR "态度"[常用字段:智能] OR "认知"[常用字段:智能] OR "需求"[常用字段:智能] OR "经验"[常用字段:智能] OR "期望"[常用字段:智能] OR "质性研究"[常用字段:智能] OR "定性研究"[常用字段:智能] OR "现象学"[常用字段:智能] OR "扎根理论"[常用字段:智能] OR "案例研究"[常用字段:智能] OR "民族志"[常用字段:智能] OR "行动研究"[常用字段:智能] OR "诠释主义"[常用字段:智能] OR "建构主义"[常用字段:智能] OR "主题分析"[常用字段:智能] OR "内容分析"[常用字段:智能] OR "焦点小组"[常用字段:智能] OR "访谈"[常用字段:智能]  **#5 #1 AND #2 AND #3 AND #4** |
| VIP | 9 | **#1** 题目或关键词："老年" OR "老年人" OR "老人"  **#2** 题目或关键词："社会隔离" OR "社会网络" OR "社会孤立" OR "社会疏离"  **#3** 题目或关键词："长期照护" OR "照护" OR "长期护理" OR "养老机构" OR "疗养院" OR "养老" OR "养老服务" OR "居家" OR "医养结合"  **#4** 题目或关键词："体验" OR "看法" OR "感受" OR "观点" OR "感知" OR "经历" OR "态度" OR "认知" OR "需求" OR "经验" OR "期望" OR "质性研究" OR "定性研究" OR "现象学" OR "扎根理论" OR "案例研究" OR "民族志" OR "行动研究" OR "诠释主义" OR "建构主义" OR "主题分析" OR "内容分析" OR "焦点小组" OR "访谈"  **#5 #1 AND #2 AND #3 AND #4** |
| Wangfang | 979 | **#1** 主题词："老年" OR "老年人" OR "老人"  **#2** 主题词："社会隔离" OR "社会网络" OR "社会孤立" OR "社会疏离"  **#3** 主题词："长期照护" OR "照护" OR "长期护理" OR "养老机构" OR "疗养院" OR "养老" OR "养老服务" OR "居家" OR "医养结合"  **#4** 主题词："体验" OR "看法" OR "感受" OR "观点" OR "感知" OR "经历" OR "态度" OR "认知" OR "需求" OR "经验" OR "期望" OR "质性研究" OR "定性研究" OR "现象学" OR "扎根理论" OR "案例研究" OR "民族志" OR "行动研究" OR "诠释主义" OR "建构主义" OR "主题分析" OR "内容分析" OR "焦点小组" OR "访谈"  **#5 #1 AND #2 AND #3 AND #4** |
